# Tipping the balance: synthesis and evaluation of centrinone-based degraders of polo-like kinase 4

**DOI:** 10.1039/d5cb00315f

**Published:** 2026-05-29

**Authors:** Andrej Kovacevic, Aleksandar Salim, Crisálida Borges, Patrick Meraldi, Sascha Hoogendoorn

**Affiliations:** a Department of Organic Chemistry, University of Geneva, 30 quai Ernest-Ansermet Geneva Switzerland sascha.hoogendoorn@unige.ch; b Department of Cell Physiology and Metabolism, University of Geneva, Rue Michel-Servet 1 Geneva Switzerland

## Abstract

Polo-like kinase 4 (PLK4) is a serine/threonine-protein kinase that plays a pivotal role in centriole biogenesis and, as such, represents a master regulator of centriole duplication. Due to its importance in cancer development and progression, PLK4 represents an attractive target for the development of novel therapeutics. Herein, we present a series of molecular degraders of PLK4, based on the highly selective PLK4 inhibitor centrinone, with the aim of targeting PLK4 for degradation *via* the ubiquitin-proteasome system. While all synthesized degraders retained low nanomolar binding affinities to the kinase domain of PLK4, large differences were found with respect to their ability to change cellular PLK4 levels. We uncover a complex pharmacological profile of the most potent degraders, D6 and D10, consisting of concomitant lowering of PLK4 levels through degradation, and enhancing PLK4 levels through inhibition of its autoregulation – dependent on its localization at the centrioles.

## Abstract 2

Bifunctional degraders based on the PLK4 inhibitor centrinone exhibit diverging cellular profiles of stabilization through inhibition and degradation through a PROTAC-mechanism – depending on the cellular localization of PLK4.

## Graphical abstract

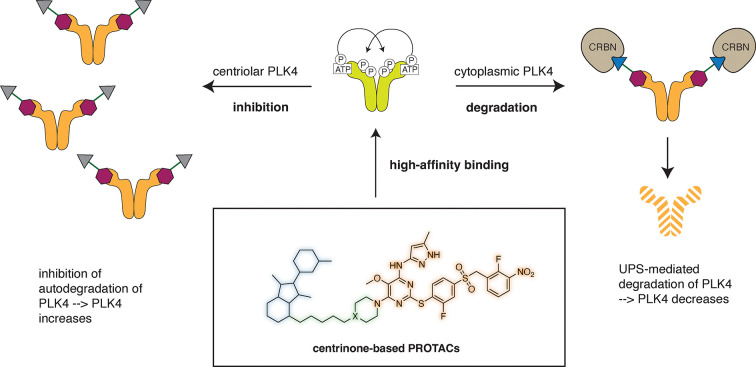

## Introduction

**Fig. 1:**
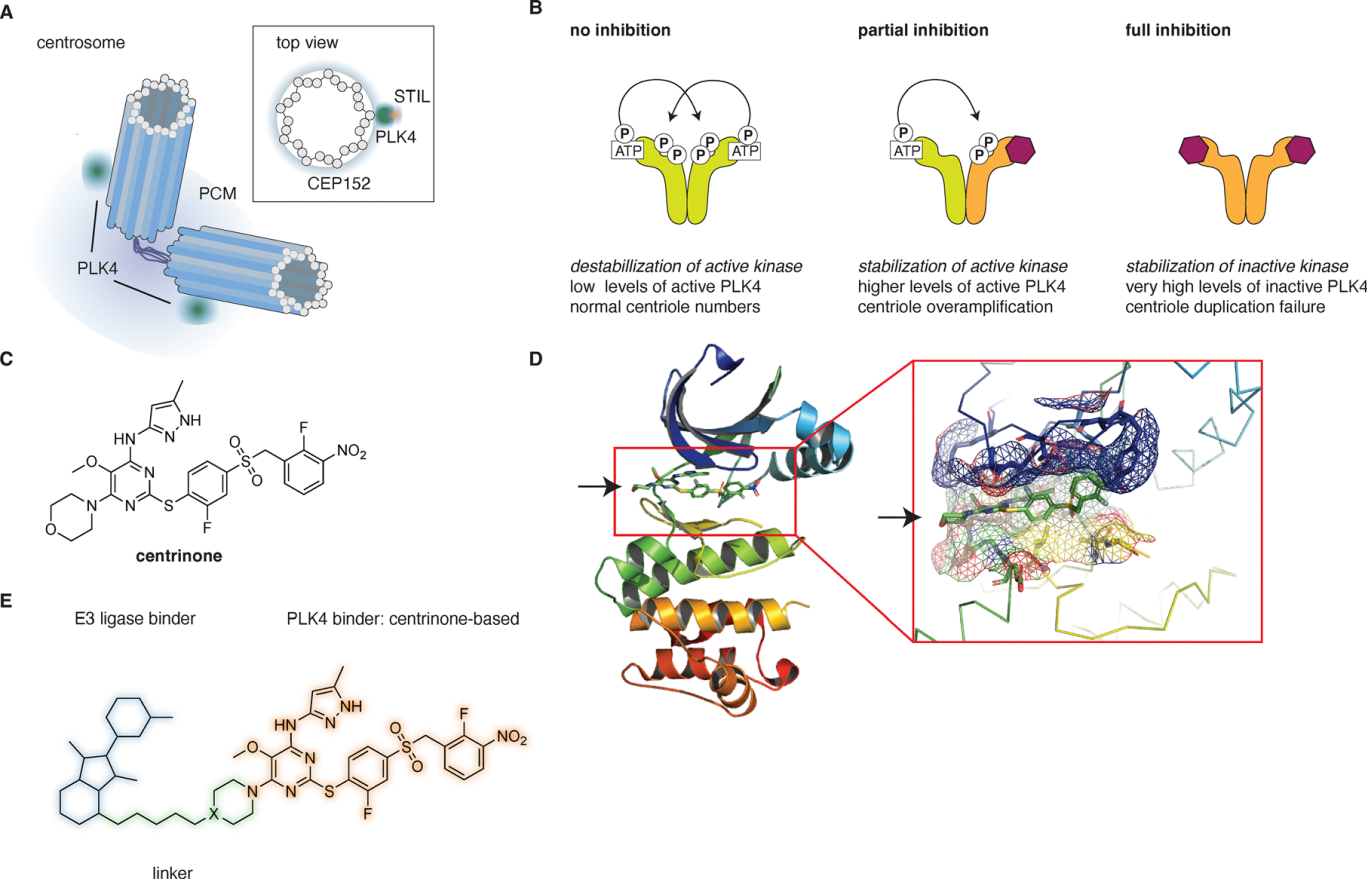
PLK4 at centrosomes and its inhibition by centrinone. (A) Schematic representation of the centrosome. (B) Cellular levels of PLK4 are tightly controlled through autoregulation, ensuring faithful centriole duplication. Partial inhibition by centrinone leads to stabilization of the active kinase and centriole overduplication, whereas full inhibition results in accumulation of inactive PLK4 and centriole duplication failure. (C) Structure of centrinone. (D) Crystal structure of centrinone bound to the PLK4 kinase domain (PDB: 4YUR). (E) General structure of centrinone-based molecular degraders.

Centrosomes (Fig. 1A) are non-membrane bound organelles comprised of two centrioles, surrounded by the pericentriolar material (PCM).^1^ Centrioles, approximately 500 nm in length and 250 nm in width, exhibit a characteristic barrel-shaped structure, arising from a nine-fold symmetrical arrangement of microtubule triplets.^2,3^ Centrosomes serve as microtubule-organizing centers and as such provide a scaffold for trafficking and organization of organelles and vesicles, while, during cell division, they facilitate microtubule spindle assembly, ensuring accurate chromosomal segregation.^4^

Proper cell division requires tight control of centriole numbers – with precisely 4 centrioles at the onset of mitosis, following a single centriole duplication event at G1/S.^5^ A protein with a critical role in this process of centriole biogenesis is polo-like kinase 4 (PLK4), a serine/threonine kinase localized at the proximal region of centrioles, on a CEP152-containing torus (Fig. 1A).^6,7^ To achieve centriole duplication rather than overamplification, activity and levels of PLK4 must be tightly regulated. PLK4 exists as a homodimer, with its activity heavily regulated by phosphorylation.^8,9^ One of the critical phosphorylation events is the trans-autophosphorylation of the degron region, leading to the recruitment of the SCFβ-TRCP E3 ligase, ubiquitination and its subsequent degradation by the proteasome, keeping the levels of PLK4 low at centrioles and preventing overamplification (Fig. 1B).^10–13^

As a key regulator of centriole duplication, any defects in the function or expression level of PLK4 can lead to detrimental consequences. Indeed, overexpression of PLK4 in mammalian cells causes centrosome amplification, promoting aneuploidy and spontaneous tumorigenesis.^14^ Furthermore, several studies have reported PLK4 overexpression in many types of cancers, with its overexpression being a biomarker for poor prognosis in patients, as well as poor response to therapy.^15–18^ Therefore, PLK4 represents an attractive therapeutic target for new chemotherapeutic drugs. Thus far, only one PLK4 inhibitor, CFI-400945, has entered Phase II clinical trials for the treatment of several malignancies.^19,20^ However, CFI-400945 lacks PLK4-selectivity,^21^ which led to the endeavours to create more selective binders, resulting in the discovery of centrinone (*K*_*i*_ = 0.16 nM), a highly selective reversible inhibitor of PLK4 (Fig. 1C).^22^ Of note, partial inhibition of PLK4 results in stabilization of the active kinase and therefore centriole overamplification, whereas full inhibition results in accumulation of the inactive kinase and centriole dilution after each cell division (Fig. 1B).^20,23,24^

Here, we aimed to take an alternative approach to classical inhibition, by converting centrinone into a proteolysis-targeting chimera (PROTAC). PROTACs are bifunctional molecules, composed of a ligand for a protein of interest (POI) covalently attached to an E3 ligase ligand *via* a small linker, and as such are capable to hijack the ubiquitin-proteasome system to induce degradation of a POI.^25–27^ In the last two decades, PROTACs emerged as powerful modalities in the treatment of various diseases, largely due to their different mode of action compared to classical inhibitors. Given the particular mode of action of centrinone, yielding high cellular levels of inactive protein, we envisioned that a centrinone-based PROTAC would provide a valuable chemical biology tool to investigate the potential roles of PLK4 beyond its main enzymatic function, which is the phosphorylation of STIL (Fig. 1A).^28,29^ We present the synthesis of 13 molecular degraders targeting PLK4 (Fig. 1E), using centrinone as the targeting ligand. The *in vitro* binding affinities of all synthesized molecules were in the low nM range, comparable to centrinone, yet large differences in cellular PLK4 levels were observed. To understand the mechanism of action we selected two distinct molecules, D6 and D10, for further evaluation, revealing an intricate balance between the intrinsic propensity of centrinone to increase PLK4 levels, and the ability of the degraders to target PLK4 for destruction, especially within the apparently shielded environment at the centrioles.

## Results

### Synthesis and *in vitro* evaluation of centrinone-based molecular degraders

**Fig. 2:**
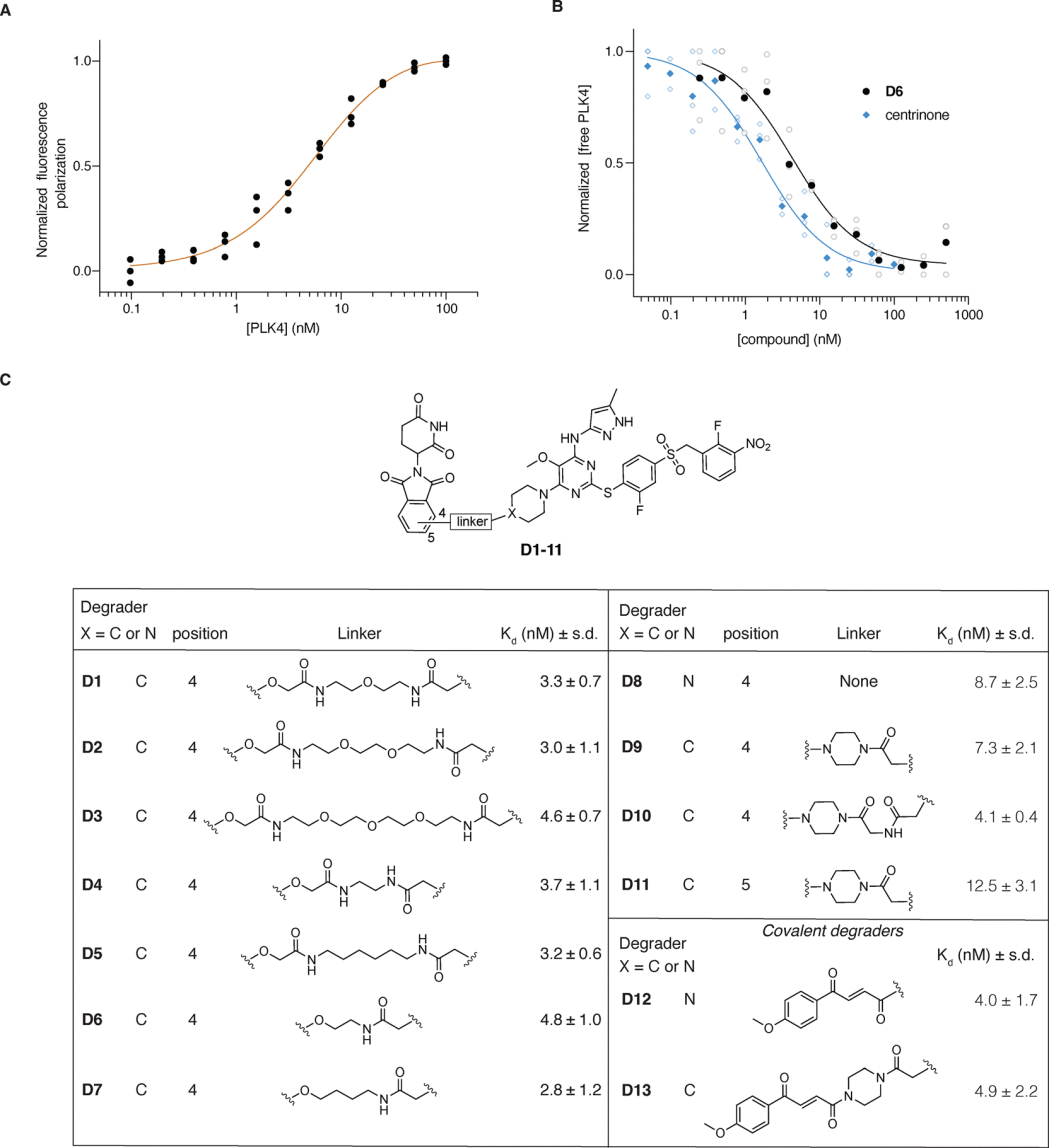
Evaluation of the binding affinities of molecular degraders to PLK4-KD using fluorescence polarization. (A) Representative binding curve of 5 nM TMR-centrinone tracer 45 to PLK4-KD (technical replicates shown, 10 independent experiments). (B) Full binding curves of centrinone (blue) and D6 (black) to PLK4-KD (10 nM; 5 nM tracer), solid symbols present the mean of 3 independent curves (open symbols). (C) Measured apparent binding affinities (mean ± s.d., *N* = 3 independent experiments) for all the synthesized PROTACs with the structures shown.

As a starting point, we analyzed the crystal structure of the PLK4 kinase domain, with centrinone bound to it (PDB: 4YUR), to identify the solvent-exposed morpholine ring as the optimal position for structural modifications (Fig. 1D).^22^ This approach was already verified in the design of fluorogenic probes which effectively target PLK4.^24^ We envisioned eleven PROTACs based on centrinone, tethered to a CRBN-binding ligand (thalidomide or its analogues) *via* linkers of varying lengths and flexibilities (Fig. 1E). Within this group, D1–7 contained flexible polyethylene-glycol or aliphatic chain linkers, while D8–D11 included more rigid piperazine-based linkers. As an alternative to CRBN targeting with thalidomide, we sought to incorporate a recently designed covalent fumarate handle, reported to target the RNF126 E3 ligase irreversibly,^30^ resulting in D12 and D13. The common centrinone precursor for all the probes, 14, containing an electrophilic chloropyrimidine moiety suitable for nucleophilic aromatic substitution, was synthesized as previously reported, with minor modifications (SI Scheme S1). Compound 14 was then converted into piperidine 16 (X = C) and piperazine 18 (X = N) (SI Scheme S2), and these were utilized for the synthesis of D1–7, D9–11 and D8, D12–13, respectively (SI Schemes S3–S9, Fig. 2C). Thalidomide-linker fragments were synthesized separately, starting either from 4-hydroxythalidomide for D1–8, or 3-/4-fluorothalidomide for D9–11 (SI Schemes S3–S8). D12–13 were obtained by regular amide coupling of phenyl-fumarate units, 40 and 42, with 16 and 18, respectively (SI Scheme S9).

To assess the binding affinities of the synthesized molecules to PLK4, we expressed and purified the 6xHis-tagged PLK4 kinase domain (PLK4-KD) and measured the dissociation constants using fluorescence polarization. For this purpose, we synthesized a fluorescent centrinone-based tracer 45 (SI Scheme S10), by coupling 16 to a tetramethylrhodamine (TMR) fluorophore *via* a short linker. When used at 5 nM, tracer 45 exhibited high-affinity binding to the purified PLK4-KD (*K*_d_ = 2.34 ± 0.80 nM, *N* = 10), comparable to centrinone (*K*_d_ = 2.26 ± 0.49 nM, *N* = 3), Fig. 2A and B). We then used this probe in a competitive binding assay to quantify the binding affinities of the PROTACs to PLK4-KD (Fig. 2B and C). We found that 5 nM tracer and 10 nM PLK4 (∼80% saturation, Fig. 2A), gave the most reproducible assay window. Satisfyingly, under these assay conditions, all of the synthesized molecules showed high binding affinities for PLK4 *in vitro*, with apparent *K*_d_ values in the nanomolar range (Fig. 2C), similar to centrinone, indicating that our structural modifications did not impact the binding to PLK4 and allowing us to proceed with in-cell experiments.

### Biological evaluation of PLK4 degradation

**Fig. 3:**
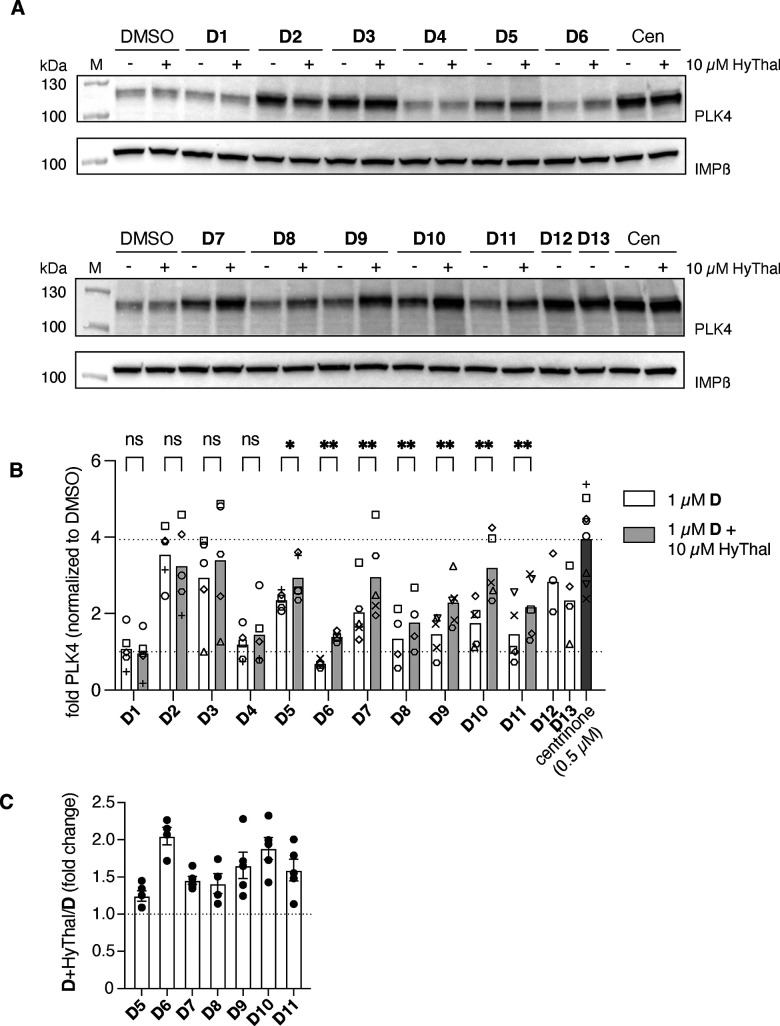
Degradation capabilities of D1–D13 (A) Representative immunoblot showing the effect of 1 µM D1–D13 on PLK4 levels in the MDA-MB-231 cell line in the presence or absence of 10 µM HyThal. IMPβ: loading control. (B) Quantification of immunoblot PLK4 band intensity of four independent experiments (symbols matched for experiment; bar represents mean). A mixed-effects model with Fisher's LSD, without multiple comparisons, was used to calculate the *p*-values between D and D + HT. *: *p* < 0.0332, **: *p* < 0.0021. (C) Ratio of PLK4 band intensities with and without 4-hydroxythalidomide (four independent experiments).

Following the fluorescence polarization results, we tested the abilities of the probes to degrade PLK4 *in cellulo*. Breast cancer MDA-MB-231 cells, which show high PLK4 expression levels, were treated with 1 µM of each of the compounds and the presence of PLK4 was probed by western blot. We determined 24 h to be an optimal time of treatment to observe effects of the molecules on PLK4 levels (SI Fig. S1). PLK4 inhibition by centrinone causes elevation of PLK4 levels, due to the inhibition of autophosphorylation, which in turn prevents its degradation (Fig. 1B and 3A). However, we found a high heterogeneity in PLK4 levels of cells treated with the degraders. Several of the synthesized probes induced PLK4 accumulation similar to centrinone (Fig. 3A, – HyThal conditions), most evident for D2, D3, D5, D7, D12 and D13, whereas others showed no or very little change compared to DMSO control (D1, D4, D6, D8). To understand this mix of behaviours, which could be the result of pure inhibition without degradation, degradation alone, or inefficient degradation/inhibition, we probed the efficacy of the probes in the presence of 4-hydroxythalidomide (HyThal). In this competition assay HyThal was used at a 10-fold excess (10 µM) to block all available CRBN E3 ligases (Fig. 3A) and prevent ternary complex formation. In this case, the sole biological effect induced by the degraders would be inhibition of PLK4, which should lead to its accumulation. Indeed, this proved true for seven of the PROTACs (D5–D11, Fig. 3B and C), with the most pronounced increase of approximately 2-fold upon HyThal co-treatment for D6 and D10 (Fig. 3C). While the fold increase for these molecules was similar, the absolute induction differed greatly. In the absence of HyThal, D6 did not induce any accumulation of PLK4, whereas D10 already showed a 1.5-fold increase compared to DMSO. Indeed, PLK4 levels in D10 + HyThal-treated cells reached those observed with centrinone, suggesting that D10 inhibits PLK4 more potently than D6, despite both compounds exhibiting similar *K*_d_ values.

**Fig. 4:**
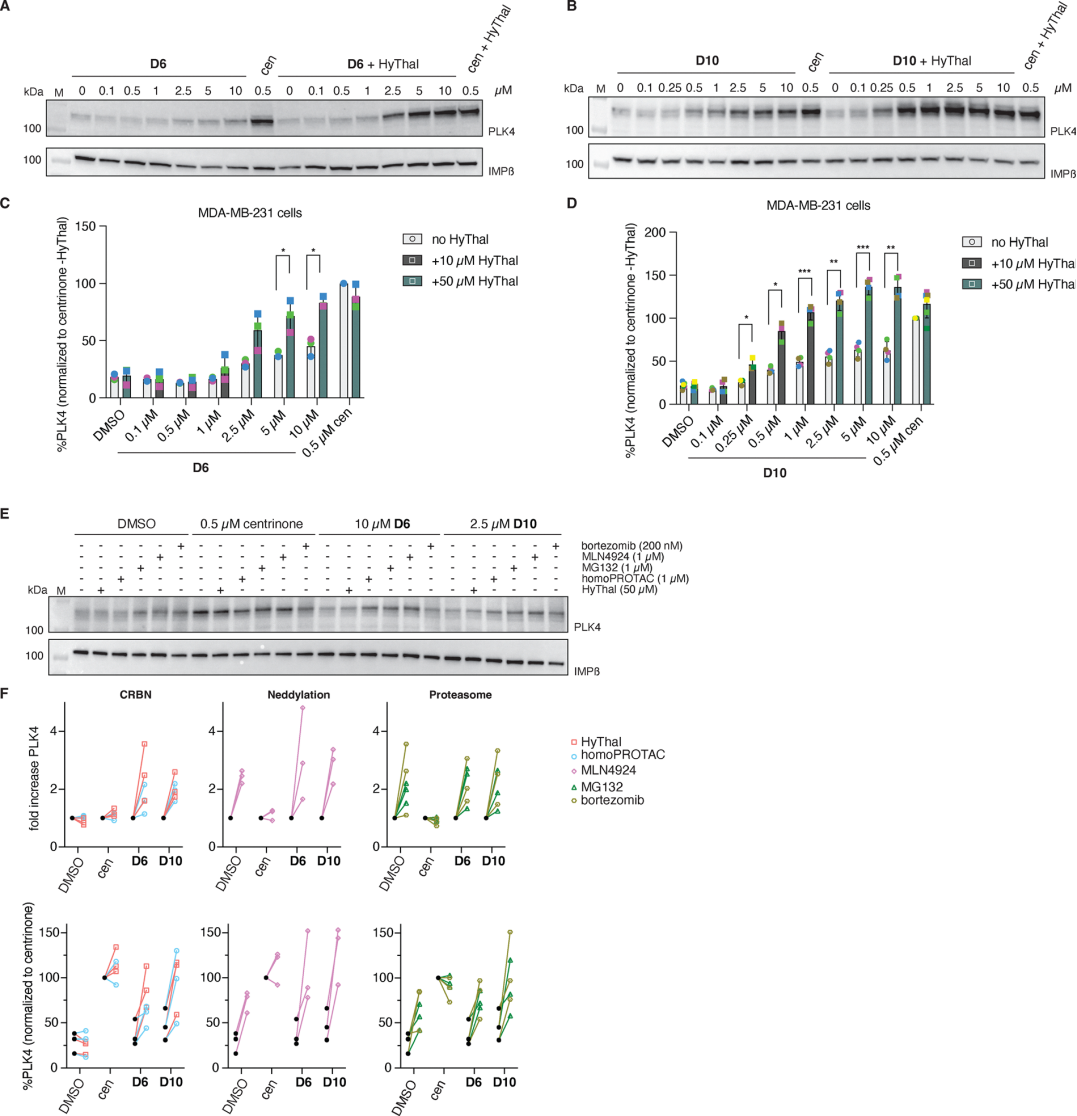
Dose-dependent changes in PLK4 levels in MDA-MB-231 cells. (A), (B) and (E) Representative immunoblots of cells treated with the indicated concentration of D6 (A) and (E) or D10 (B) and (E) in the presence or absence of the indicated competitor concentrations. IMPβ: loading control (C) and (D) Bargraphs showing the PLK4 quantification of 3 (C) or 4 (D) independent experiments (symbols). Bars represent means. Unpaired *t*-tests, *: *p* < 0.0332, **: *p* < 0.0021, ***: *p* < 0.0002 (E) representative immunoblot of 3 independent experiment is shown, with quantification of the fold induction and relative PLK4 amounts of each experiment shown in the lineplots in (F).

We therefore selected both D6 and D10 for further investigation, as both showed prominent degradation, with D6 appearing as a poor inhibitor and D10 showing potent inhibition at 1 µM. To determine their *in cellulo* potencies, we tested D6 and D10 in a range of concentrations (10 µM to 100 nM) in both MDA-MB-231 and HeLa (cervical cancer) Centrin1-GFP^31^ cell lines in the absence or presence of an excess of HyThal (Fig. 4 and SI Fig. S2). These experiments revealed several things. First, while western blot quantification of low levels of PLK4 proved not accurate enough to obtain statistical significance for all comparisons, in both cell lines, and for both compounds, there was a clear upward trend with HyThal competition, indicative of compound-induced degradation of PLK4 (Fig. 4A–D and SI Fig. S2).

The ability of both compounds to inhibit, and thus accumulate PLK4, was stronger in HeLa cells (SI Fig. S2) compared to MDA-MB-231 cells (Fig. 4), which may be attributed to differences in basal levels of PLK4 between these cells and/or cell permeability affecting intracellular compound concentrations. Second, D10 was the more potent inhibitor of the two, resulting in accumulation of PLK4 in HeLa cells starting at the 0.25 µM concentration, whereas for D6 about 10-fold more compound was needed to detect an increase in PLK4 by western blot. These results highlight the complexity of the biology induced by both D6 and D10 and that the resulting PLK4 levels are a product of two concurrent inseparable events: inhibition and degradation.

To further prove that both D6 and D10 act as PROTACs, we performed a series of competition experiments with inhibitors targeting various proteins involved in CRBN-mediated proteasomal degradation, including a CRBN inhibitor and degrader (HyThal and a CRBN homoPROTAC^32^), proteasome inhibitors (MG132 and bortezomib), as well as the neddylation inhibitor MLN4924, which inhibits NEDD8 that has a crucial role in the activation of E3 ligase complexes (Fig. 4E and F).^33–35^ Indeed, all of these co-treatments increased PLK4 levels in degrader-treated cells compared to degrader alone, whereas none of the treatments could further increase the high levels of inactive PLK4 caused by centrinone inhibition. In contrast, MLN4924 or proteasome inhibitors caused about a 2-fold elevation of PLK4 levels in DMSO-treated cells. Indeed, endogenous PLK4 levels are highly regulated through ubiquitination by neddylation-dependent SCFβ-TRCP, and subsequent proteasomal degradation.^12,33^ No effect of HyThal or the CRBN-targeting homoPROTAC (resulting in chemical knockdown of CRBN, SI Fig. S3^32^) was found on DMSO-treated cells, further strengthening that CRBN does not play a role in the endogenous PLK4 homeostasis. In addition, we examined whether D6 and D10 caused degradation of GSPT1, a known neosubstrate of thalidomide-based degraders and its analogues and observed no degradation of GSPT1 (SI Fig. S3).^36^

Since it is not possible to distinguish the distribution of uninhibited, partially, and fully inhibited PLK4 by western blot, band intensities may not be reflective of the induced phenotypes. To assess this, we performed centriole scoring experiments. The recruitment of PLK4 to centrioles is strictly regulated, and it is known that centrinone inhibition of PLK4 leads to prevention of procentriole formation, resulting in centriole depletion in dividing cells. Contrastingly, partial inhibition, by lower concentrations of centrinone, causes centriole overamplification, through inhibition of degradation-inducing autophosphorylation (Fig. 1B).^20,23,24^ We treated HeLa centrin-GFP cells with varying concentrations of D6 and D10 in the presence and absence of HyThal and counted the number of centrioles present after 24 h of treatment (SI Fig. S4). In accordance with the inhibitory potencies (SI Fig. S2), D10 treatment resulted in more potent centriole depletion compared to D6, whereas for D6 centriole overamplification was the predominant phenotype. No significant differences were found in the induced phenotypes in the presence of HyThal for the stronger inhibitor D10. For D6, at the 5 µM concentration there was a slight shift from overamplification to centriole depletion in the presence of HyThal, indicative of stronger inhibition in the absence of degradation. To exclude the possibility that this was the result of a higher effective concentration of the probe (as no E3 ligase is bound), rather than a reflection of a shift in degradation/inhibition, we sought to further confirm the ability of D6 and D10 to degrade PLK4 in a more direct manner.

### D6 and D10 potently degrade an autoregulation-deficient PLK4 mutant and non-centriolar endogenous PLK4

**Fig. 5:**
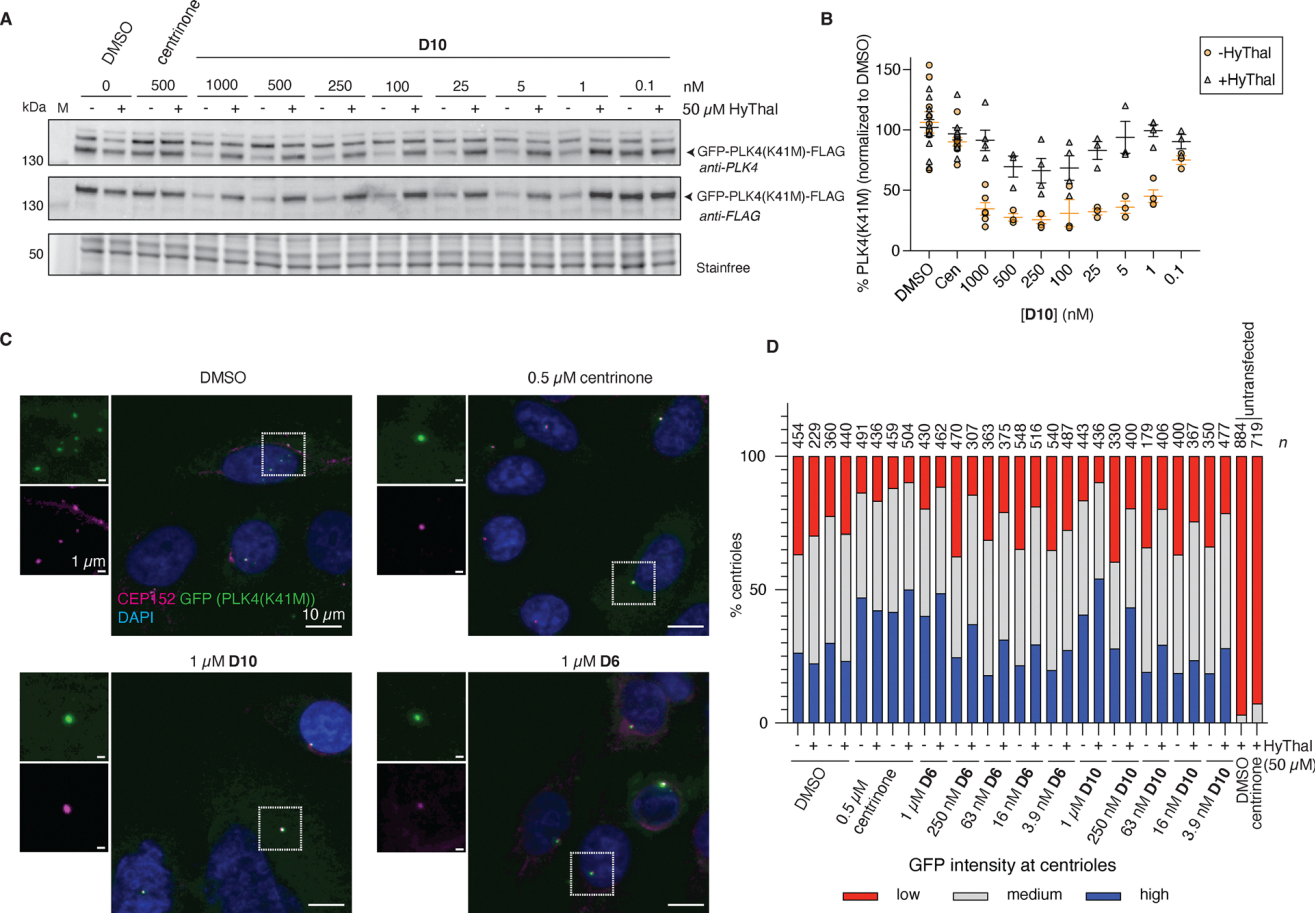
GFP-PLK4(K41M)-FLAG and non-centriolar PLK4 are degraded by D6 and D10. (A) and (B) HeLa cells overexpressing GFP-PLK4(K41M)-FLAG were treated with the indicated concentration of D10 with or without 50 µM HyThal for 24 h and probed for the presence of PLK4 and FLAG by western blot. (A) representative WB and (B) quantification of PLK4 immunoblots of N ≥ 3 independent experiments (indicated by symbol), line and error bars indicate mean ± SEM. (C) Representative immunofluorescence images and (D) quantification of GFP intensity at the centrioles of HeLa cells expressing GFP-PLK4(K41M)-FLAG. n = number of centrioles analyzed, data shown is from one out of two independent experiments.

As increasing levels of PLK4 upon inhibition prevented a proper assessment of the degradation capacities of D6 and D10, we overexpressed a mutant PLK4, PLK4(K41M), that is incapable of autoregulation in HeLa cells, preventing the complex phenotype as observed with endogenous PLK4.^10^ We observed very potent degradation of overexpressed GFP-PLK4(K41M)-FLAG by both D6 and D10 by western blot (Fig. 5A, B and SI Fig. S5A, B). However, degradation plateaued at ∼30% PLK4(K41M) remaining, and so we turned to fluorescence microscopy to understand where the remaining PLK4 was localized.

As overexpression was done transiently, we observed high heterogeneity in expression levels, with a small percentage (typically ∼5%) of highly transfected cells having predominant cytoplasmic GFP signal, and lower expressing cells showing both cytoplasmic and centriolar GFP signal. We found that the overall percentage of highly overexpressing cells was reduced by D10, in line with the western blot result (SI Fig. S5C), which could be competed with HyThal to levels observed with centrinone. Of note, centrinone-treated cells also had fewer bright cells than DMSO control, because of stringent masking criteria in the analysis, excluding bright round cells. However, for centrinone, in contrast to D10, this effect was independent of the presence of HyThal and likely reflects a mild toxicity. As expression levels found by western blot for DMSO and centrinone (Fig. 5A and B) treatments were similar, highly transfected cells likely contributed greatest to the overall signal detected by blot, and we assume that those cells are more easily washed away during immunofluorescence protocols, compared to direct lysis of the cells for western blot. At all concentrations tested, we could observe a dot of PLK4 at the centrioles, both using a PLK4 antibody (detecting mutant and endogenous PLK4, SI Fig. S5D) and a GFP antibody (solely detecting mutant PLK4, Fig. 5C and D). The intensity of PLK4 at centrioles increased using centrinone, or high (1 µM) concentration D6 or D10 in the presence of HyThal, consistent with inhibition and stabilization of endogenous PLK4 (SI Fig. S5D). The fraction of GFP positive centrioles also increased under the same conditions, consistent with a model where endogenous PLK4 can phosphorylate and thereby destabilize the mutant, which is counteracted by inhibitor treatment (Fig. 5D).^10,12^ While the difference between with and without HyThal indicates that there is some degradation occurring which prevents levels at the centrioles from increasing in the absence of HyThal, similar to what we found for overall amounts of endogenous PLK4 by western blot (Fig. 4 and SI Fig. S2), these levels did not go below that found for DMSO-treated cells. Together, this raises the possibility that a large proportion of PLK4 at the centrioles is in some way shielded to small molecule-mediated degradation, yet not to inhibition. This would also provide an explanation as to why there was little to no phenotypic shift observed upon HyThal treatment in the centriole scoring experiment (SI Fig. S4).

**Fig. 6:**
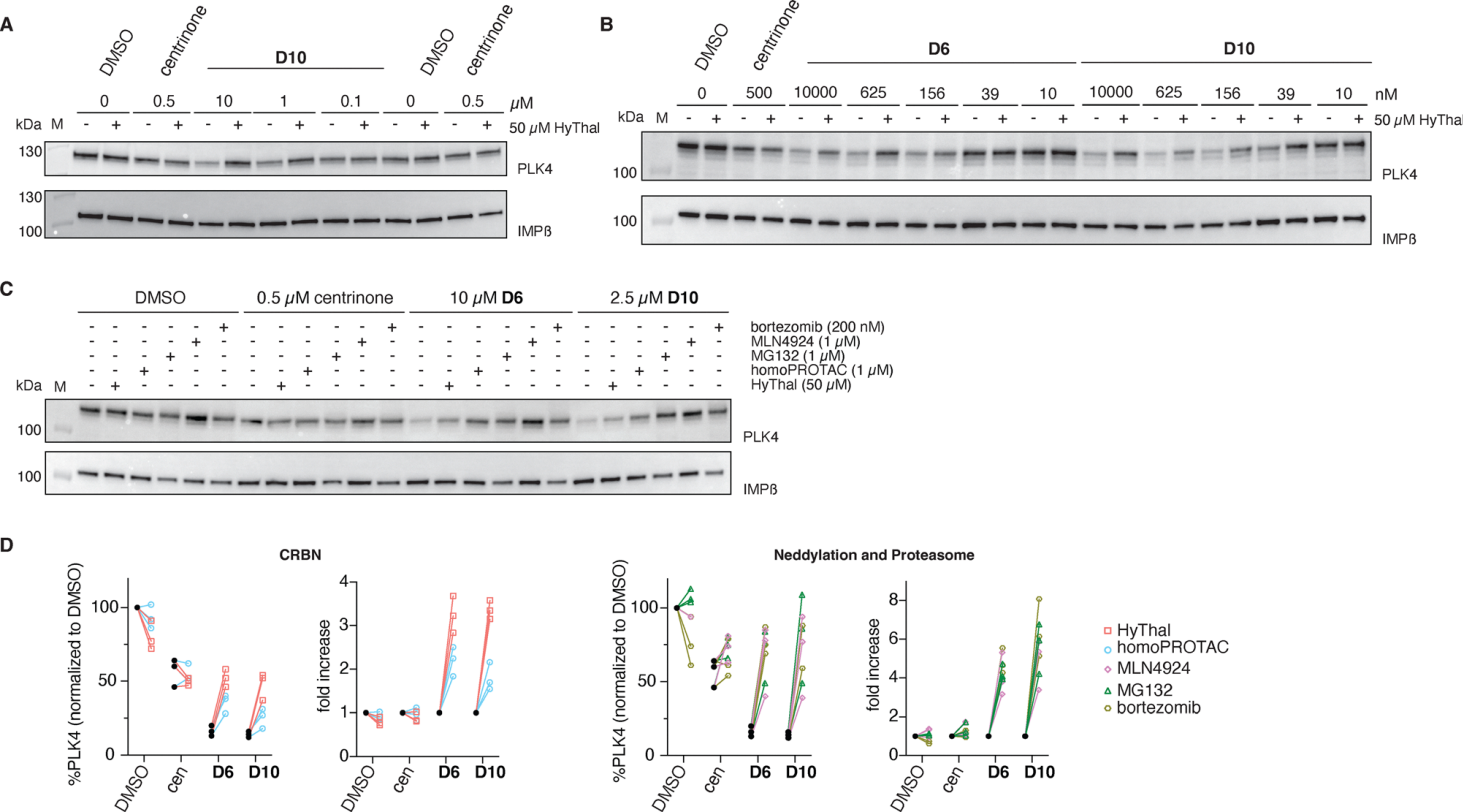
D6 and D10 are potent degraders of acentriolar PLK4. (A) Acentriolar RPE-1 USP28^−/−^ cells were treated with DMSO, 500 nM centrinone or compound D10 with or without 50 µM HyThal and the degradation of endogenous PLK4 was assessed by western blot. Representative blot of two independent experiments. (B) Acentriolar RPE-1 USP28^−/−^ SAS6^−/−^ cells were treated with DMSO, centrinone, and the indicated concentrations of D6 or D10 with or without 50 µM HyThal and the degradation of endogenous PLK4 was assessed by western blot. Representative blot of three independent experiments. (C) RPE-1 USP28^−/−^ SAS6^−/−^ cells were treated with DMSO, centrinone, D6 or D10 with or without the indicated concentrations of competitors and PLK4 levels were assessed by western blot. A representative blot of three independent experiments is shown, which were quantified in (D) for relative PLK4 levels and fold change. IMPβ: loading control.

To further test this hypothesis, we next used RPE-1 (non-transformed retina pigment epithelial cells) USP28^−/−^ cells, that were chronically treated with centrinone to fully deplete centrioles while remaining cycling-competent (USP28 is part of a mitotic surveillance mechanism that arrests the cell cycle of cells lacking centrosomes^37^). Because of chronic centrinone treatment, we found that the PLK4 levels in these cells are relatively high, enabling detection by western blot, yet its localization is non-centriolar as the cells lack centrioles. We found that PLK4 levels quickly decreased to barely detectable levels upon centrinone washout (SI Fig. S6A), and for that reason cells were maintained with centrinone until the start of the experiment, where they were switched to medium without centrinone after a single wash. Under these experimental conditions it is likely that low concentrations of centrinone remained present inside the cells, preventing the centrioles from re-forming and keeping PLK4 levels stable. Indeed, 24 h after washing out centrinone, DMSO-, centrinone- or D10-treated cells were still acentriolar (as judged by CEP152 staining, SI Fig. S6B). PLK4 levels were stable between DMSO- or centrinone-treated cells but decreased in D10- or D6-treated cells in a dose-dependent manner (Fig. 6A and SI Fig. S6C). Protein levels could be fully restored in the presence of HyThal, illustrating CRBN-dependent degradation of endogenous PLK4 induced by D6 and D10. Importantly, no conclusions about degradation potency should be drawn in this experimental setup, as the starting point is centrinone-bound PLK4, which requires an additional displacement step before the degraders can bind.

To overcome this limitation, we genetically removed centrioles through knockout of SAS6 in RPE-1 USP28^−/−^ cells (SI Fig. S7). In a *Drosophila* study, PLK4 was found to be more stable, yet inactive, in SAS-6 knock-down cells.^13^ In line with that, we found PLK4 to be readily detectable in RPE-1 USP28^−/−^ SAS6^−/−^ cells (Fig. 6B). We subjected the cells to increasing concentrations of D6 and D10 and observed high degradation potency of both compounds, reaching maximum degradation of PLK4 at concentrations as low as 156 nM (Fig. 6B and SI Fig. S6D). However, similar to experiments with the PLK4(K41M) mutant (Fig. 5A, B), degradation plateaued at ∼20%, which we hypothesize to be due to elements of the pericentriolar matrix, which are still present in acentriolar cells (SI Fig. S7) and could be shielding PLK4 from complete degradation. Protein levels could be restored to centrinone-treated levels in the presence of HyThal, illustrating CRBN-dependent degradation of endogenous PLK4 induced by D6 and D10 (Fig. 6B–D). Additionally, PLK4 could be rescued by proteasome inhibitors (MG132 and bortezomib), the neddylation inhibitor MLN4924 and the CRBN homoPROTAC (Fig. 6C and D), further substantiating that the observed degradation is indeed caused because D6 and D10 act as PROTACs. In striking contrast to rising PLK4 levels in centriolar cells observed upon centrinone, MLN4924, or proteasome inhibitor treatment (Fig. 4E), we observed lower PLK4 levels upon centrinone treatment (not affected by CRBN homoPROTAC nor HyThal), and no effect on PLK4 levels by proteosome or neddylation inhibitors in DMSO-treated cells (Fig. 6C and D), indicating that endogenous acentriolar PLK4 is not autoregulated by autophosphorylation in the same manner as centriolar PLK4. Together, these experiments conclusively prove the ability of the PROTACs to degrade endogenous PLK4 in the absence of centrioles.

## Discussion

The selective PLK4 inhibitor centrinone has enabled the controlled depletion of centrioles from mammalian cells, but, through its mechanism of action, leads to high cellular levels of inhibited PLK4. Here, we sought to leverage the high affinity binding and selectivity of centrinone to prepare a library of molecular degraders that would enable cellular PLK4 depletion. Towards the same goal, Sun *et al.* recently synthesized a PROTAC,^38^ named SP27, based on another PLK4 inhibitor, CZS-034. Surprisingly, while CZS-034 has been reported as a type I (ATP-competitive)-inhibitor, like centrinone, no PLK4 stabilization has been shown for this compound.^39^ To the best of our knowledge, there is no experimental evidence why this is the case, but possibly the off-rate for this compound is fast, enabling occasional auto-phosphorylation. SP27-mediated PLK4 degradation was potent but also incomplete. A detailed mechanistic investigation of SP27 or CZS-034 on centrioles, or PLK4 localization to centrioles, has not been performed, in contrast to the well-studied effects of centrinone on PLK4 stability and centriole duplication. Therefore, we decided to employ centrinone as the ligand of choice for our PROTAC design. Satisfyingly, our synthesized centrinone-based degraders retained low nanomolar affinity for the kinase domain of PLK4, illustrating that the scaffold is tolerant to chemical changes, in agreement with what has been described for a centrinone-based fluorescent probe.^24^ Upon cellular evaluation, however, we found large differences between the different degraders, ranging from no effect, to pure inhibition and selective degradation. These differences are likely due to factors such as cell permeability (for inactive compounds such as D1 or D4) and the ability of the compounds to engage in ternary complex formation between PLK4 and CRBN (for pure inhibitors such as D2 and D3). While this was not investigated here, cellular engagement assays such as nanoBRET for CRBN or HiBit-based ternary complex assays could be used to assess these different behaviours in detail.^40,41^ For the two most potent degraders, D6 and D10, we discovered a cellular phenotype resembling that of centrinone, but especially for D10, the balance between inhibition and degradation remained strongly at inhibition. To circumvent the autoregulation of PLK4, a phosphorylation deficient mutant provided the means to conclusively prove the ability of these compounds to degrade PLK4. Under the conditions tested, we could not, however, degrade endogenous PLK4 beyond basal levels, nor could we remove PLK4(K41M) from the centrioles, prompting us to hypothesize that PLK4 at the centrioles may be shielded from degradation. The different capacity of D6 and D10 to inhibit PLK4 further illustrates that there may be differential accessibility for these compounds to PLK4 at the centrioles, as both compounds were equally potent in their degradation of a cytoplasmic PLK4 mutant and showed the same binding affinity to purified PLK4 *in vitro*. PLK4 at centrioles has been shown to have strong binding interactions with both scaffolding proteins (CEP152)^6,7^ and phosphorylation substrates (STIL).^28,29^ Centrinone binding, while preventing the PLK4-STIL interaction, does not interfere with the PLK4-CEP152 interaction, but rather increases the number of interaction sites to 9 over prolonged exposure times.^42^ Moreover, Scott *et al.* have shown that centrinone-mediated inhibition of PLK4 leads to much reduced turnover time (and thus accumulation) of PLK4 at centrioles, more so than was observed with inhibition of proteosomal degradation.^42^ Possibly, this PLK4-CEP152 interaction blocks the accessibility to CRBN – a requirement for functional PROTAC-mediated degradation. For example, Gasic *et al.* have shown that tubulin is able to evade CRBN-targeted degradation, conceivably because of its association with other proteins in tubulin dimers or microtubules.^43^ Satisfyingly, we were able to conclusively show that endogenous PLK4 could be degraded in acentriolar cells, strengthening the hypothesis that PLK4 at centrioles is shielded. However, even in the absence of centrioles, degradation was incomplete. For acentriolar cells generated through chronic centrinone treatment, this could be due to the imperfect experimental system, which necessitates a competition between the probes and centrinone. In addition, it is unknown if the cytoplasmic PLK4 retains some level of interaction with scaffolding proteins even in the absence of centrioles, which could be an alternative explanation of incomplete degradation. Indeed, for acentriolar cells generated through SAS6 depletion, we could demonstrate the presence of centriolar matrix proteins, which could explain why degradation did not go beyond 80%. Taken together, we envision that our probes will be valuable tools to further decipher the accessibility of PLK4, especially when combined with genetic perturbation of key PLK4 interactors or centriole components. Moreover, our data confirm that endogenous PLK4 levels are sensitive to neddylation and proteosome inhibitors in centriolar cells, in line with SCFβ-TRCP-mediated ubiquitination upon autophosphorylation. While SCFβ-TRCP PROTACs so far consist of cell-impermeable, peptide-based SCFβ-TRCP ligands, recent studies have reported small-molecule binders that hold promise for cell-permeable SCFβ-TRCP-based PROTAC development.^44^ Recently, the ubiquitin ligase CRL4DCAF1 was shown to be important for PLK4 regulation, and as such this E3 ligase is an interesting target to explore for the development of next-generation PLK4-targeting PROTACs with possibly different capacities for ternary complex formation and degradation at the centrioles.^45,46^

## Conclusions

In this study, we report the successful synthesis of a series of molecular degraders of PLK4, with low nanomolar *in vitro* binding affinities. PROTACs D6 and D10, while both able to degrade PLK4, differed severely in their ability to inhibit PLK4, with D6 being a poor inhibitor and D10 a strong inhibitor. Both compounds were nanomolar potent degraders of a PLK4 mutant and endogenous PLK4 in acentriolar RPE-1 cells. Together, our study uncovers that PLK4 accessibility at centrioles can be limited for targeted degraders, enabling binding and inhibition, but largely preventing degradation. Changing the PLK4-targeting motif beyond type-1 inhibitors as well as engaging alternative E3 ligases are considered promising future directions to shift the balance towards PLK4 degradation at centrioles.

## Author contributions

Conceptualization: A. S., S. H.; formal analysis: A. S., A. K., S. H.; funding acquisition: S. H., P. M.; investigation: A. K., A. S., C. B.; resources: P. M., S. H.; supervision: A. S., P. M., S. H.; visualization: A. S., S. H.; writing – original draft: A. K., S. H.; writing – review & editing: A. K., A. S., C. B., P. M., S. H.

## Conflicts of interest

There are no conflicts to declare.

## Supplementary Material

CB-OLF-D5CB00315F-s001

## Data Availability

The raw experimental data that support the findings of this study are available in Zenodo with the identifier https://doi.org/10.5281/zenodo.17738398. Supplementary information (SI): supplementary Fig. S1–S7, supplementary Schemes S1–S10, full experimental procedures (biology and chemistry), uncropped western blots, and analytical data (NMR spectra). See DOI: https://doi.org/10.1039/d5cb00315f.
